# Modifiable Resources and Resilience in Racially and Ethnically Diverse Older Women: Implications for Health Outcomes and Interventions

**DOI:** 10.3390/ijerph19127089

**Published:** 2022-06-09

**Authors:** Sparkle Springfield, Feifei Qin, Haley Hedlin, Charles B. Eaton, Milagros C. Rosal, Herman Taylor, Ursula M. Staudinger, Marcia L. Stefanick

**Affiliations:** 1 Department of Public Health Sciences, Parkinson School of Health Sciences and Public Health, Loyola University Chicago, Maywood, IL 60153, USA; 2 Department of Medicine, Stanford Center for Biomedical Informatics Research, Quantitative Sciences Unit, Stanford University, Palo Alto, CA 94304, USA; fqin@stanford.edu (F.Q.); hedlin@stanford.edu (H.H.); 3 Department of Epidemiology, Brown University School of Public Health and Department of Family Medicine, Warren Alpert Medical School of Brown University, Providence, RI 02912, USA; cbeaton51@gmail.com; 4 Department of Population and Quantitative Health Sciences, Medical School, University of Massachusetts, Worcester, MA 01605, USA; milagros.rosal@umassmed.edu; 5 Cardiovascular Research Institute Research, Morehouse School of Medicine, Morehouse College, Atlanta, GA 30310, USA; htaylor@msm.edu; 6 Technische Universität Dresden (TUD), 01069 Dresden, Germany; rektorin@tu-dresden.de; 7 Department of Medicine, Stanford Prevention Research Center, Stanford University, Palo Alto, CA 94305, USA; stefanick@stanford.edu

**Keywords:** resilience, race/ethnicity, resources, aging, women’s health, Women’s Health Initiative

## Abstract

**Introduction**: Resilience—which we define as the “ability to bounce back from stress”—can foster successful aging among older, racially and ethnically diverse women. This study investigated the association between psychological resilience in the Women’s Health Initiative Extension Study (WHI-ES) and three constructs defined by Staudinger’s 2015 model of resilience and aging: (1) perceived stress, (2) non-psychological resources, and (3) psychological resources. We further examined whether the relationship between resilience and key resources differed by race/ethnicity. **Methods**: We conducted a secondary analysis on 77,395 women aged 62+ (4475 Black or African American; 69,448 non-Hispanic White; 1891 Hispanic/Latina; and 1581 Asian or Pacific Islanders) who enrolled in the WHI-ES, which was conducted in the United States. Participants completed a short version of the Brief Resilience Scale one-time in 2011. Guided by Staudinger’s model, we used linear regression analysis to examine the relationships between resilience and resources, adjusting for age, race/ethnicity, and stressful life events. To identify the most significant associations, we applied elastic net regularization to our linear regression models. **Findings**: On average, women who reported higher resilience were younger, had fewer stressful life events, and reported access to more resources. Black or African American women reported the highest resilience, followed by Hispanic/Latina, non-Hispanic White, and Asian or Pacific Islander women. The most important resilience-related resources were psychological, including control of beliefs, energy, personal growth, mild-to-no forgetfulness, and experiencing a sense of purpose. Race/ethnicity significantly modified the relationship between resilience and energy (overall interaction *p* = 0.0017). **Conclusion**: Increasing resilience among older women may require culturally informed stress reduction techniques and resource-building strategies, including empowerment to control the important things in life and exercises to boost energy levels.

## 1. Introduction

When “resilience” first appeared in theories of developmental child psychology, it was characterized as an intrinsic personal characteristic [1,2]. Now, it is generally regarded as a dynamic constellation that improves over time as one successfully navigates and “bounces back” from life stressors [3,4]. While leading evidence suggests that resilience plays an integral role in successful aging [5,6,7], relatively little is known about the factors that are associated with resilience among aging populations—particularly racially and ethnically diverse groups of women in the United States. This gap is particularly important to fill, considering growth in the older population (including increases in the proportion of racial/ethnic minorities) and growing concern about society’s ability to meet the health needs of this aging population with sufficient socioeconomic and psychological resources to improve and sustain quality of life [8,9].

Higher self-reported psychological resilience (hereafter resilience) has been significantly associated with better mental and physical health outcomes among vulnerable populations [10,11,12,13,14,15,16,17,18,19]. Yet, a growing body of evidence suggests that race-related stress (e.g., racism) can undermine resilience [9,20]. African American women, and to a lesser extent other women of color, are more likely to experience elevated levels of individual and environmental stressors across the life course compared to non-Hispanic White women; in some studies, these disparities persist after adjustment for socioeconomic factors [21,22,23,24]. Hence, evaluating a variety of resources and resilience in the context of racial/ethnic differences may be an important first step in informing the development of intervention strategies to address health disparities and promote health equity in older populations.

Indeed, medical literature has highlighted sociodemographic [25,26], psychosocial [7,25,26,27], and physical characteristics associated with successful aging [17,27,28,29]. For example, in a small subset of predominantly White women enrolled in the WHI at the San Diego Clinical Center (*n* = 1395, 76% White), Lamond and colleagues reported that higher emotional well-being, optimism, self-rated successful aging, social engagement, and fewer cognitive complaints were most strongly associated with resilience [27]. Building upon this work, we are characterizing resilience in the larger WHI sample. Despite evidence suggesting that social identity (e.g., race/ethnicity, gender) significantly influences resilience, there are limited data on the potential interaction between resilience and race/ethnicity in older women [30,31,32,33,34]. Accordingly, we investigated whether associations between resources and resilience varied as a function of race/ethnicity in older women (aged 62+) based at 40 sites across the United States.

We explored the association between resilience, as measured by an adapted version of the Brief Resilience Scale (BRS), and three constructs defined by Staudinger’s (2015) resilience model, including: (1) perceived stressors, (2) non-psychological resources (e.g., absence of major illness, physical functioning, annual family income, education, and marital status), and (3) psychological resources (i.e., personal growth, control of beliefs, a sense of purpose, memory, self-reported energy, and social support). Staudinger’s theory defines resilience as a constellation of risk factors (stressors) and protective resource factors (both of a psychological and non-psychological nature). Such that if one can mobilize enough available resources, they can successfully navigate their stressors to achieve (the maintenance of) subjective well-being. In this way, the three constructs of stressors, non-psychological and psychological resources, work together to demonstrate the capacity of an individual to bounce back from stress. We hypothesized that both non-psychological and psychological resources would be significantly associated with resilience while controlling for perceived stressors and that race/ethnicity would modify these relationships, given our theoretical model and the broader literature [35,36,37].

Ultimately, this paper seeks to determine the resources most strongly associated with resilience, and how the relationships between these resources and resilience vary by race/ethnicity. Our findings highlight both non-psychological (e.g., socioeconomic) and psychological (e.g., control of beliefs) resources that may be used to understand and potentially intervene in resilience among older, racially, and ethnically diverse groups of women.

## 2. Methods

### 2.1. Design and Study Population

The Women’s Health Initiative (WHI) is a long-term national health study that focuses on strategies for preventing common causes of morbidity and mortality in older women. Participants were recruited from areas surrounding 40 clinical centers established primarily at major academic health centers across the United States, including urban, suburban, and rural populations. Written informed consent was obtained from 161,808 postmenopausal women, ages 50–79 years, between 1993 through 1998 (baseline) through institutional review boards at one of these clinical centers across the United States to participate in either one to three randomized, controlled trials or an observational study, through 2005. At that time, most active participants (*n* = 115,407) provided written consent to continue to be followed for an additional five years at their respective clinical centers. In 2010, most still active study participants (*n* = 93,567) provided written consent to ongoing follow-up in the WHI Extension Study (WHI-ES) by the WHI Clinical Coordinating Center (Fred Hutchinson Cancer Research Center, Seattle, WA, USA).

The current study is a cross-sectional analysis of all WHI-ES participants who completed the Brief Resilience Scale, which was offered one-time in 2011 on survey form 155, along with stress and resource measures described below. Paper surveys were mailed to participants’ homes and completed with a pencil. Women were not compensated. Women who identified as American Indian or Alaskan Native or categorized as “Other” were excluded from our main analysis due to their low numbers (American Indian or Alaskan Native *n* = 241, 0.3%; Other *n* = 739, 0.9%, respectively). Our decision to exclude the American Indian or Alaskan Native women from the analysis was driven by the small number of women in the low resilience category (*n* = 23). Many of the stressors and resources we considered are categorical, and we expected to have zero or low number cells for several of the variables considered (e.g., only 24% of women did not have a stressful life event, 6% had living assistance, income and education had 4+ levels). Small numbers and zero cells prevent models from converging, producing unstable results. Considering the limited epidemiological data on American Indian or Alaskan Native women in the literature, we performed a post hoc descriptive analysis to provide information on perceived stress and resilience in the results section.

### 2.2. Measures

**Figure 1:**
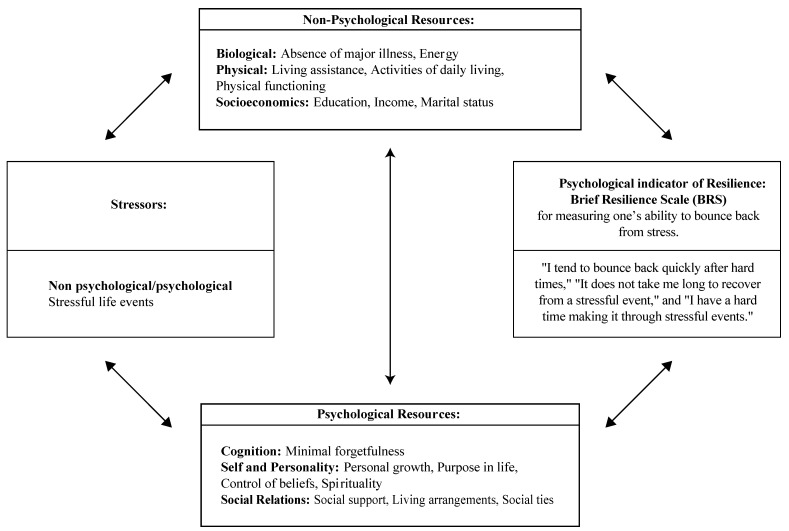
Stressors, resources (non-psychological and psychological), and self-reported psychological resilience [adapted from Staudinger’s theoretical model focused on resilience and aging (Staudinger et al. 2015)].

Except for potential modifiers, such as age and race/ethnicity, all exposure variables were organized into Staudinger’s theoretical model focused on resilience and aging (2015) [3], as described in the introduction. This approach revealed how perceived stress and resources (non-psychological and psychological) might work together to explain the ability to bounce back from stress in women enrolled in the WHI-ES (see Figure 1 below). Here, we elaborate on the theory’s origins and foundations.

Staudinger’s theoretical model defines resilience as a relational construct, a constellation of stressors (risk factors) on the one hand and accessibility of protective factors on the other, to gain a better understanding of how these factors result in a specific developmental outcome (e.g., subjective well-being, resilience) [38]. These resilience constellations are dynamic and highly specific as they may vary across time between groups or with an individual depending on the situation and outcome being considered. The primary resilience constructs examined in the present study (e.g., stressors, resources) are not static or mutually exclusive. The main foundations of Staudinger’s model include lifespan developmental (examination of human behavior across one’s entire lifespan) and systems psychology (synthesis of biological, social, cultural, and environmental contextual factors). Notably, Staudinger’s theoretical framework can serve as a model for mediation analyses to test whether the associations between stressors and indicators of well-being are transmitted via specific resources. However, these analyses are not performed in the present study. 

Our study variables (including primary outcome measure of resilience and three constructs defined by Staudinger’s 2015 model of resilience and aging: (1) perceived stress, (2) non-psychological resources, and (3) psychological resources) were self-reported on WHI form 155 which was collected in 2011, except for baseline socio-demographic (e.g., age, race/ethnicity, education, income, marital status) and health variables (e.g., diagnosis of stroke, diabetes, all cancers (except skin), lupus, Parkinson’s disease, and Alzheimer’s disease). Detailed descriptions of all study measures appear in Supplemental Table S1. See brief descriptions below.

To assess resilience, participants rated the following statements (adapted from the Brief Resilience Scale (BRS)) using a 5-point Likert scale (strongly disagree/agree): “I tend to bounce back quickly after hard times”, “It does not take me long to recover from a stressful event,” and “I have a hard time making it through stressful events” [39]. Note that the BRS measure was taken one-time (on WHI form 155) in 2011. The alpha coefficient for the three items was 0.74, suggesting that the items have relatively high internal consistency. While this could be a limitation—the three items measure the core resilience construct because it focuses on one’s ability to bounce back from stress. 

We used the stressful life events measure to capture perceived stressors in WHI-ES participants, consisting of 12 questions collected in 2011. For example, women were asked to recall whether they had experienced a stressful life event in the past year and, if so, how much it had upset them. Follow-up options ranged from 0 (never) to 3 (very much). These questions were based on a life change measure for the Beta-Blocker Heart Attack Trial [40].

Data on resource variables, both non-psychological and psychological, were organized by domains in Staudinger’s theoretical model. Non-psychological resources and corresponding variable domains included: biological (e.g., absence of major illness, energy—subscale of quality of life), physical (e.g., living assistance, activities of daily living), and socioeconomics (e.g., level of education, annual household income, marital status). Psychological resource domains included: cognition (e.g., minimal forgetfulness), self and personality (e.g., personal growth, purpose in life, control of beliefs, spirituality—because it is a self-regulatory process), and social relations (e.g., social support, living arrangements, social ties) [39,40,41,42,43,44,45]. In addition to self-reported resource measures, the absence of major illness was determined using a combination of adjudicated and self-reported outcome data on the presence of illnesses such as stroke, diabetes, and cancer. See Figure 1 and Supplemental Table S1 for the complete list of domains and variable descriptions.

During the 1993–1998 WHI baseline visits, participants completed questionnaires that asked about age (years) and race/ethnicity (Black or African American, non-Hispanic White, Hispanic/Latina, Asian or Pacific Islanders, American Indian or Alaskan Native, and Other). Additionally, note that our study intentionally uses the term “race/ethnicity” (instead of ethnicity alone) throughout the paper because race (e.g., Black, White) was included in the classification used during the original data collection. Therefore, it may be a valuable marker of social, cultural, and historical experience [40,41,42]. Furthermore, this study aligns with the approach to race as a “social construction” and “cultural driver of history,” which denotes the consequences of identity politics (e.g., race-related stress, access to health-related resources) and the lived experience of our participants. In this way, the term “race/ethnicity” may be helpful to the interpretation of our findings.

### 2.3. Statistical Analysis

Sociodemographic and other characteristics of the cohort were summarized using means and standard deviations for continuous variables and count and percent for categorical variables. The percentages displayed were based on the number of participants with non-missing data for each variable. Although we treat resilience as a continuous outcome in our model building models (Table 1), for ease of interpretation, we display in Table 2 resilience levels by suggested cutoffs for low (0.1–2.9), medium (3.0–4.2), and high (4.3–5.0) [46].

To evaluate the association between resilience and the various psychological and other resources laid out above, we ran a series of traditional multivariable linear regression models, adjusting for sequentially added covariates. Linear regression was used to fit separate models adjusted for each individual resource variable on its own, adjusted for age (Model 1), adjusted for age and race/ethnicity (Model 2), and adjusted for age, race/ethnicity, and stressful life events (Model 3) in addition to a set of crude models that were not adjusted for any covariates. Then to identify the variables most strongly associated with resilience, we used elastic net regularization for variable selection and ranked the selected variables based on their importance [47]. Elastic net variable selection was applied to three linear models that included all non-psychological and psychological variables along with age, race/ethnicity, and stressful life events (Model E1); non-psychological variables only (Model E2); psychological variables only (Model E3) in addition to age, race/ethnicity, and stressful life events in all three models. Elastic net regularization allowed us to rank the variables (using variable importance) in an unbiased manner that handled correlation between the variables in a way that would not penalize highly correlated variables [47]. Standard variable selection methods such as univariate or backward selection have been shown to be biased. 

To evaluate potential race/ethnicity differences in the relationship between resilience and the resource variables; we fit separate linear regression models with an added interaction term between race/ethnicity and the top five resource variables identified from elastic net regularization (Models 4–8; see Supplemental Table S3) [48]. The results are displayed as plots illustrating the interaction effects. The panel plot for Models 4, 7, and 8 is shown below (as they have statistically significant interaction terms), and the Model 5 and Model 6 plots are shown as Supplemental Figures S1 and S2 with full details on Models 4–8 displayed in Supplemental Table S3). We tested whether the interaction was statistically significant using a joint hypothesis test comparing the interaction model (i.e., a model with interaction terms between each race/ethnicity indicator variable and the resource variable) to the model with no interaction terms.

To address missing data in the cohort, we used multiple imputations by chained equations (MICE) to create 5 imputed datasets that were then combined using Rubin’s rules. The ‘type3_MI_glm’ macro developed by Wang et al. was used to calculate the overall effect of the interaction term in Models 4–8 [49]. All analyses were performed using SAS (version 9.4; SAS Institute Inc., Cary, NC, USA) and R (version 3.5.2; R (version 3.5.2; R Foundation for Statistical Computing, Vienna, Austria). All tests were two-sided and evaluated at an alpha level of 0.05.

## 3. Results

Our study analytic sample included 77,395 women enrolled in WHI-ES with a mean age (±standard deviation) of 76.99 (±6.4) years and following self-identified race/ethnicity: Black or African American (*n* = 4475, 5.8%), Hispanic/Latina (*n* = 1891, 2.4%), Asian or Pacific Islander (*n* = 1581, 2.0%), non-Hispanic White (*n* = 69,448, 89.7%). See Table 2. Most women self-reported medium (*n* = 36,638, 47.3%) or high (*n* = 35,261, 45.6%) levels of resilience. On average, all women had low levels of perceived stress [stressful life events (mean ± standard deviation) 3.20 ± 3.13 out of 36; higher score indicates greater perceived stress]. Similarly, in the post hoc analysis, American Indian or Alaskan Native women self-reported low levels of perceived stress [stressful life events (mean ± standard deviation) 4.04 ± 3.69 out of 36; and low (*n* = 23, 9.5%), medium (*n* = 110, 45.6%) and high (*n* = 108, 44.8%) levels of resilience.

In the main analysis, Black or African American women reported the highest resilience, followed by Hispanic/Latina women, Asian or Pacific Islander, and White women. Note, we also calculated the proportion of women who reported a high level of resilience within each racial/ethnic group, including American Indian or Alaskan Native women, which also suggested that Black or African American women (*n* = 2174, 48.6%) reported the highest levels of resilience, followed by non-Hispanic White (*n* = 31,584, 45.5%), American Indian or Alaskan Native (*n* = 108, 44.8%), Asian (*n* = 684, 43.3%) and Hispanic/Latina women (*n* = 819, 43.3%). On average, women who reported higher resilience also had fewer stressful life events and more resources. As for psychological resources, women who reported high resilience had minimal forgetfulness and higher personal growth, purpose, control of beliefs (i.e., how often have you felt that you were unable to control the essential things in your life?), spirituality, social support, and social ties compared to women who reported low resilience. With respect to non-psychological resources, women who reported high resilience tended to have no major illnesses, higher energy levels to do things, no need for living assistance, more independence in performing activities of daily living, increased physical functioning, a 4-year college degree, annual family income of USD75,000+, and were not divorced or separated.

As demonstrated in Table 1 (fully adjusted Model 3), most of the relationships between resilience and the resources were statistically significant after adjusting for age, race/ethnicity, and perceived stress. Compared to women with low resilience, women with high resilience were more likely to: be younger, African American, have lower perceived stress, have fewer major illnesses, have higher energy, not reside in assisted living facilities (e.g., nursing homes), be independent in performing activities of daily living, have greater physical functioning, have higher levels of education, have higher annual family income, and be single. Marital status was the only resource that showed no statistically significant association with resilience.

**Figure 2:**
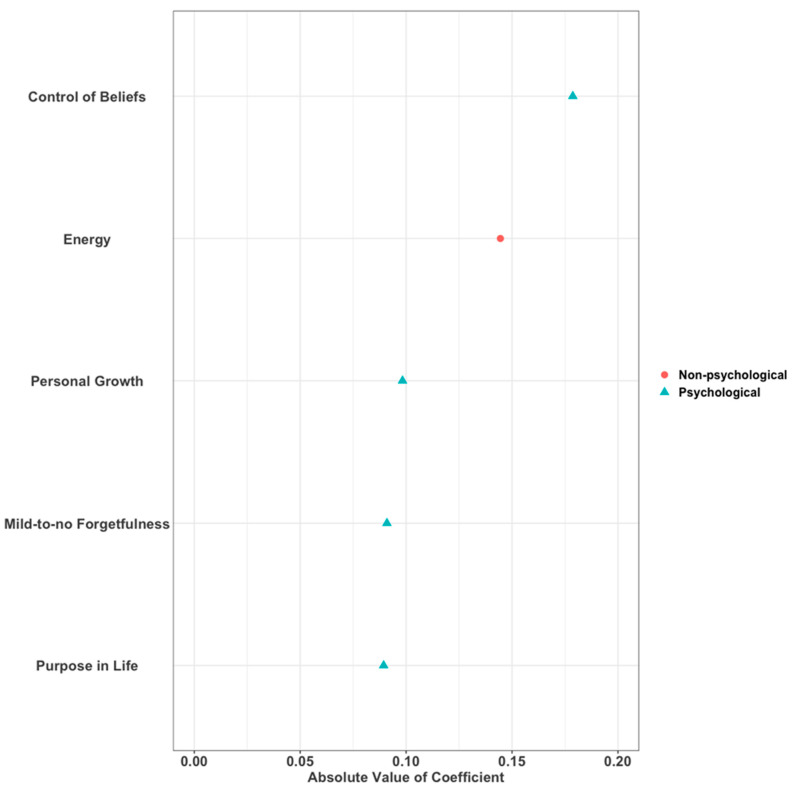
Ranking of variable importance in Elastic Net model using all variables (Top 5 variables from Model E1 shown).

We used elastic net regularization applied to our linear regression models to identify the most important resource factors (both non-psychological and psychological) associated with resilience. See Figure 2 and Supplemental Table S2a–c. Overall, control of beliefs, energy, personal growth, mild-to-no forgetfulness, and purpose in life ranked as the most influential resources based on Model E1. Figure 2 illustrates the ranking of all resources resulting from the variable selection method, and Supplemental Table S2a lists the corresponding coefficient estimates. In terms of non-psychological resources, energy, a 4-year college degree, annual family income of USD75,000+, and independence in performing activities of daily living were ranked as the top resources for resilience based on Model E2. Supplemental Figure S2 illustrates the ranking of non-psychological resources (only), and Supplemental Table S2b lists the corresponding coefficient estimates. Finally, control of beliefs, life purpose, personal growth, social support, and mild-to-no forgetfulness ranked as the top psychological resources for resilience based on Model E3. Supplemental Figure S3 illustrates the ranking of psychological resources (only), and Supplemental Table S2c lists the corresponding coefficient estimates.

**Figure 3:**
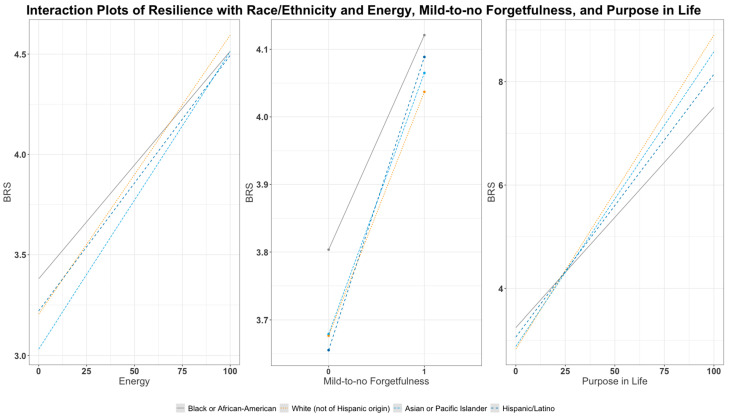
Plot displaying interaction effects between race/ethnicity and energy score, mild-to-no forgetfulness, and purpose in life score as estimated from Model 4, Model 7, and Model 8.

With a joint hypothesis test, we tested for race/ethnicity differences in the associations between resilience and the top five resource factors—energy (Model 4), control of beliefs (Model 5), personal growth (Model 6), mild-to-no forgetfulness (Model 7), and purpose in life (Model 8). See Models 4, 7, and 8 in Figure 3. These associations differed by racial/ethnic groups with respect to energy (overall *p*-value for interaction = 0.0017), mild-to-no forgetfulness (overall *p*-value for interaction = 0.0028), and purpose in life (overall *p*-value for interaction = 0.0023) but not control of beliefs (overall *p*-value for interaction = 0.089) and personal growth (overall *p*-value for interaction = 0.15; Supplemental Table S3 and Figure 3). The results indicated that Asian or Pacific Islanders women experienced the greatest increase in resilience with each unit increase in energy score (estimate: 0.015; 95% CI: 0.011–0.019), followed by non-Hispanic White (estimate: 0.014; 95% CI: 0.0136–0.0142), Hispanic/Latina (estimate: 0.013; 95% CI: 0.010–0.015), and Black or African American women (estimate: 0.011; 95% CI: 0.0089–0.014). The association of mild-to-no forgetfulness with resilience was the highest among non-Hispanic White women, where those with minimal forgetfulness had a 0.43 unit increase in resilience (estimate: 0.43; 95% CI: 0.42–0.45) compared to those who experienced more forgetfulness. Hispanic/Latina women with minimal forgetfulness had a 0.39 unit increase in resilience (estimate: 0.39; 95% CI: 0.29–0.48) compared to those with more forgetfulness followed by Asian or Pacific Islander women (estimate: 0.36; 95% CI: 0.24–0.48), and Black or African American women (estimate: 0.32; 95% CI: 0.25–0.38). Similarly, non-Hispanic White women also showed the largest association of resilience with each unit increase in purpose in life score (estimate: 0.061; 95% CI: 0.056–0.065) followed by Asian or Pacific Islander (estimate: 0.057; 95% CI: 0.045–0.069), Hispanic/Latina (estimate: 0.051; 95% CI: 0.021–0.081), and Black or African American women (estimate: 0.043; 95% CI: 0.0064–0.079).

## 4. Discussion

Guided by Staudinger’s 2015 theoretical model, which focused on aging and resilience, this study investigated the association between resilience and resource factors (both non-psychological and psychological in nature). After adjusting for stressful life events, age, and race/ethnicity, the strongest associations were control of beliefs, energy, personal growth, mild-to-no forgetfulness, and life purpose. Importantly, the relationship between resilience and energy was significantly modified by race/ethnicity. The magnitude of this relationship was lowest among Black or African American women (hereafter African American), even though they reported the highest levels of resilience compared to other racial/ethnic groups. This may imply that African American women have to expend significantly more energy to achieve the same level of resilience as their non-African American counterparts. Also, our findings lend support to defining resilience as an outcome—i.e., a result of successfully navigating exposure to high-stress situations. Our theoretical model suggests resilience is a constellation of resources and stressors, not an internal force of personality traits [3]. In any case, once an individual identifies their stressors and resources, they require the belief that they can take control of the situation and the energy to navigate them [50]. Our findings suggest these resource variables and cultural values are important to resilience in older racially and ethnically diverse women in the United States.

This study is one of the first investigations to demonstrate racial/ethnic differences in resilience in a large and exclusive cohort of older women. We observed that African American women have the strongest positive relationship with resilience. Our findings may point toward the unique socio-cultural and historical context in which African American women live as they are consistently dealing with at least two forms of oppression, racism, and sexism [51,52]. Furthermore, additional chronic stressors have stemmed from race-based oppression (i.e., anti-Black racism), such as decreased access to health resources in African American communities [53,54,55]. It follows that constant navigation of these chronic stressors and daily stressors (i.e., perceived discrimination) may contribute to an advanced capability to seek out resources and develop resilience among our sample of older African American women [56,57,58]. Conversely, evidence suggests that these stressful experiences may significantly influence African American women’s stress appraisal and coping mechanisms. As a result, they may report higher rates of resilience than they are actually experiencing (e.g., Superwomen role) [59]. Still, in recent literature, both individual and collective resilience is cited as central to African American women’s historical legacy and continued survivorship [34,60,61,62].

Our findings cohere with previous literature that suggests both non-psychological and psychological resources are essential to cultivating and maintaining resilience throughout the aging process [3,35,36], particularly control of beliefs and energy. Research suggests that most of the top five resource factors, including energy, personal growth, mild-to-no forgetfulness, and life purpose, can be improved through behavioral and lifestyle interventions [63,64,65]. The psychological resources have been characterized in the literature and our theoretical model as “personality strengths” that can be used to promote positive adjustments, embodied thoughts, feelings, and behaviors [3,66]. For example, coaching and educational interventions can have a positive influence on one’s personal growth and life purpose [67]. Evidence suggests control of beliefs significantly varies over the aging process, and subsequently, more work is needed to develop strategies to enhance control of beliefs in older populations [68]. Although it will not always be possible to modify this factor, researchers can (and should) take these beliefs into account when developing resilience-based interventions [68]. The resource factors that are not clearly psychological (e.g., energy and mild-to-no forgetfulness) may also be modifiable through lifestyle interventions (e.g., diet, physical activity, and sleep) [69]. Physical activity and cognition (e.g., problem-solving training, mnemonic training, and guided imagery) interventions in older women are also valuable [65].

The relationship between resilience and energy was significantly modified by race/ethnicity. Even though they reported the highest levels of resilience, the magnitude of this relationship was lowest among African American women. Fortunately, evidence suggests that increased resilience can lead to increased energy. For example, a study conducted among health professionals (working in stressful workplaces) who engaged in a resilience intervention training—including mindfulness, gratitude, and forgiveness exercises—revealed that had increased psychological resilience, they felt better prepared to handle stress, a renewed sense of energy, and had overall improvements in well-being [70]. This may suggest that increased energy levels are particularly crucial to fostering resilience in vulnerable sub-populations. Still, the relatively weak relationship between resilience and energy may suggest the need for further research focused on identifying the most relevant resources for African American women.

This study is not without limitations. The primary outcome measure was an adapted short version of the Brief Resilience Scale, consisting of only three items. Though limited in number, these three items measure the core resilience construct by focusing on one’s ability to bounce back from stress. Moreover, the alpha coefficient was 0.74, suggesting that the items have relatively high internal consistency. While we do not measure discrimination, substantial evidence suggests that racial/ethnic subgroups are socially constructed and are rough approximations of exposure to racism [71,72]. Similarly, we did not have a measure of control of beliefs. Instead, those questions derived from the perceived stress scale items focused on perceived control.

This study is also limited by its cross-sectional design. Guided by a theoretical framework, the direction of the relationship is implied for interpretability, albeit we recognize that all observed relationships are bi-directional. Further research to empirically establish the directionality of the relationships and the role of potential mediators is needed. Our theoretical model notes potential overlap (misclassification) between Staudinger’s constructs and the non-psychological psychological resource factors with bidirectional arrows. We responded to this limitation by including all types of resources in the linear regression models and elastic net regularization analysis. Additionally, our theoretical model is designed to test how resources mediate the relationship between stressors and developmental outcomes. However, as an initial step, our study uses this framework to guide the examination of the relationship between perceived stressors, resources (non-psychological and psychological), and resilience during a single period. 

Future studies may build on our work, including more time points, conducting mediation analyses, and providing further insights into the potential mechanism by which women enrolled in the WHI-ES bounce back from stress. Lastly, all the resource variables are self-reported and adapted measures. Additional studies are warranted to examine the relationship between resilience and evidence-based resource measures—particularly concerning control of beliefs, energy, and cognition among older racially and ethnically diverse women in the United States.

## 5. Conclusions

To our knowledge, this is among the first studies of its kind to focus on measuring resilience among older racially and ethnically diverse women in the United States. Guided by the resilience and aging theoretical model, we employed a sophisticated variable selection methodology (elastic net regularization) to highlight the most important resource variables to resilience in our population. Ultimately, we examined a variety of non-psychological and resilience resources, several of which are modifiable and ought to be further examined to inform the development of intervention strategies to improve resilience in older women. Of further value, we examined racial/ethnic interactions to provide evidence on cultural differences between resilience and resource factors. These can be further investigated in future resilience studies targeting specific racial/ethnic groups, such as African American women. Not only are a variety of resource factors significantly and positively associated with resilience, but those also that are most strongly associated with resilience are modifiable and can be useful foundations for interventions. 

Stress reduction, resource-building strategies, practices that boost energy levels, as well as the assessment of control of beliefs over the essential things in life may help increase resilience and related skills among older, racially and ethnically diverse women. Leveraging the resilience of African American women may be a key strategy to improve health outcomes in their communities, as well as addressing racial and ethnic health disparities. This study takes a foundational step toward formative intervention research focused on building resilience resources with respect to cultural differences between racial/ethnic groups. Our findings can also be used to raise awareness of important resource variables to resilience among stakeholders (e.g., people with lived experience, not for profit research organizations, researchers, clinician-scientists, policymakers) as well as inform implementation science methods (e.g., train the trainer, champions, dissemination via public education sessions/clinic brochures).

## Figures and Tables

**Table 1:** Associations between age, race/ethnicity, stressors, resources (non-psychological and psychological), and self-reported psychological resilience from participants in the Women’s Health Initiative Extension Study. Note, each row shown corresponds to an individual fitted model.

Covariates	Crude	Model 1 Age-Adjusted	Model 2 Age and Race/Ethnicity Adjusted	Model 3 Age, Race/Ethnicity and Stressor Adjusted
		Estimate (95% CI)	Estimate (95% CI)	Estimate (95% CI)
**POTENTIAL** **MODIFIERS**				
Age (per year)	−0.0099 (−0.011, −0.0090); *p* < 0.0001			
Race/ethnicity	Joint *p*-value = 0.0056	Joint *p*-value = 0.0008		
non-Hispanic White	−0.031 (−0.056, −0.0064)	−0.011 (−0.036, 0.013)		
Asian or Pacific Islander	−0.039 (−0.086, 0.0081)	−0.032 (−0.079, 0.014)		
Black or African American	Reference	Reference		
Hispanic/Latina	−0.077 (−0.12, −0.033)	−0.084 (−0.13, −0.041)		
**STRESSORS**				
Stressful life events	−0.047 (−0.049, −0.045); *p* < 0.0001	−0.049 (−0.051, −0.047); *p* < 0.0001	−0.049 (−0.051, −0.047); *p* < 0.0001	
**RESOURCES**				
**non-psychological**				
**Physical**				
Absence of major illness (y/n)	−0.13 (−0.14, −0.11); *p* < 0.0001	−0.11 (−0.13, −0.093); *p* < 0.0001	−0.11 (−0.13, −0.093); *p* < 0.0001	−0.089 (−0.11, −0.072); *p* < 0.0001
Energy score	0.015 (0.014, 0.015); *p* < 0.0001	0.015 (0.0143, 0.0151); *p* < 0.0001	0.015 (0.014, 0.015); *p* < 0.0001	0.014 (0.013, 0.014); *p* < 0.0001
Living Assistance (y/n)	−0.24 (−0.27, −0.22); *p* < 0.0001	−0.20 (−0.22, −0.17); *p* < 0.0001	−0.20 (−0.22, −0.18); *p* < 0.0001	−0.18 (−0.20, −0.15); *p* < 0.0001
Activities of Daily Living score	0.071 (0.064, 0.077); *p* < 0.0001	0.062 (0.056, 0.069); *p* < 0.0001	0.062 (0.056, 0.069); *p* < 0.0001	0.060 (0.054, 0.066); *p* < 0.0001
Physical Functioning score	0.0061 (0.0058, 0.0063); *p* < 0.0001	0.0060 (0.0057, 0.0062); *p* < 0.0001	0.0060 (0.0057, 0.0063); *p* < 0.0001	0.0055 (0.0052, 0.0057); *p* < 0.0001
**Socio-economic status**				
Education	Joint *p*-value < 0.0001	Joint *p*-value < 0.0001	Joint *p*-value < 0.0001	Joint *p*-value < 0.0001
≤HS	Reference	Reference	Reference	Reference
HS or GED	0.13 (0.084, 0.18)	0.13 (0.080, 0.17)	0.13 (0.078, 0.17)	0.11 (0.065, 0.15)
Vocational or training school/some college orassociate degree	0.22 (0.17, 0.27);	0.21 (0.16, 0.25)	0.20 (0.16, 0.25)	0.19 (0.15, 0.23)
≥College degree	0.30 (0.25, 0.34)	0.28 (0.23, 0.32)	0.28 (0.23, 0.32)	0.24 (0.20, 0.29)
Annual Family Income	Joint *p*-value < 0.0001	Joint *p*-value < 0.0001	Joint *p*-value < 0.0001	Joint *p*-value < 0.0001
<20,000	Reference	Reference	Reference	Reference
20,000–34,999	0.085 (0.063, 0.11);	0.081 (0.059, 0.10)	0.082 (0.059, 0.10)	0.072 (0.050, 0.094)
35,000–49,999	0.14 (0.12, 0.16);	0.13 (0.10, 0.15)	0.13 (0.10, 0.15)	0.11 (0.086, 0.13)
50,000–74,999	0.19 (0.17, 0.22);	0.17 (0.14, 0.20)	0.17 (0.15, 0.20)	0.15 (0.13, 0.18)
75,000+	0.26 (0.24, 0.29);	0.23 (0.20, 0.25)	0.23 (0.21, 0.26)	0.20 (0.18, 0.23)
Married or living with partner, (y/n)	−0.0015 (−0.014, 0.011); *p* = 0.82	−0.015 (−0.028, −0.0027); *p* = 0.017	−0.015 (−0.027, −0.0025); *p* = 0.019	−0.0015 (−0.014, 0.011); *p* = 0.81
**Psychological**				
**Cognition**				
Mild-to-no Forgetfulness, (y/n)	0.49 (0.47, 0.50); *p* < 0.0001	0.47 (0.46, 0.49); *p* < 0.0001	0.47 (0.46, 0.49); *p* < 0.0001	0.42 (0.41, 0.44); *p* < 0.0001
**Self and Personality**				
Personal Growth score	0.056 (0.047, 0.066); *p* < 0.0001	0.056 (0.046, 0.066); *p* < 0.0001	0.056 (0.048, 0.065); *p* < 0.0001	0.054 (0.046, 0.062); *p* < 0.0001
Purpose in Life score	0.061 (0.050, 0.072); *p* < 0.0001	0.061 (0.050, 0.073); *p* = 0.0001	0.061 (0.051, 0.072); *p* < 0.0001	0.059 (0.048, 0.069); *p* < 0.0001
Control of Beliefs score	0.17 (0.167, 0.173); *p* < 0.0001	0.168 (0.165, 0.171); *p* < 0.0001	0.168 (0.165, 0.171); *p* < 0.0001	0.16 (0.15, 0.16); *p* < 0.0001
Spirituality score	0.042 (0.040, 0.044); *p* < 0.0001	0.042 (0.040, 0.044); *p* < 0.0001	0.043 (0.041, 0.045); *p* < 0.0001	0.043 (0.041, 0.045); *p* < 0.0001
**Social Relations**				
Social Support score	0.028 (0.025, 0.030); *p* < 0.0001	0.027 (0.025, 0.029); *p* < 0.0001	0.027 (0.025, 0.029); *p* < 0.0001	0.025 (0.023, 0.027); *p* < 0.0001
Living Arrangements score	−0.021 (−0.035, 0.0069); *p* = 0.006	−0.038 (−0.050, −0.026); *p* < 0.0001	−0.037 (−0.050, −0.025); *p* < 0.0001	−0.025 (−0.036, −0.015); *p* < 0.0001
Social ties score	0.039 (0.036, 0.042); *p* < 0.0001	0.037 (0.034, 0.040); *p* < 0.0001	0.037 (0.034, 0.040); *p* < 0.0001	0.035 (0.032, 0.038); *p* < 0.0001

**Table 2:** Demographics, stressors, resources (non-psychological and psychological) by self-reported psychological resilience from participants in the Women’s Health Initiative Extension Study.

	Total	Low (BRS 1.0–2.9)	Medium (BRS 3.0–4.2)	High (BRS 4.3–5.0)
CHARACTERISTICS	*n* = 77,395	*n* = 5496 (7.1%)	*n* = 36,638 (47.3%)	*n* = 35,261 (45.6%)
**POTENTIAL MODIFIERS**				
Age at data collection on resilience in years (mean (sd))	76.99 (6.41)	77.67 (6.69)	77.37 (6.44)	76.49 (6.29)
Race/ethnicity (%)				
Black or African American	4475 (5.8)	387 (7.0)	1914 (5.2)	2174 (6.2)
non-Hispanic White	69,448 (89.7)	4840 (88.1)	33,024 (90.1)	31,584 (89.6)
Hispanic/Latina	1891 (2.4)	177 (3.2)	895 (2.4)	819 (2.3)
Asian or Pacific Islander	1581 (2.0)	92 (1.7)	805 (2.2)	684 (1.9)
**STRESSORS**				
**non-psychological**				
Have had stressful life events over the past year (%)	Missing *n* = 293	Missing *n* = 46	Missing *n* = 144	Missing *n* = 103
No	18,597 (23.8)	1108 (19.9)	7610 (20.6)	9879 (27.7)
Yes	59,638 (76.2)	4450 (80.1)	29,421 (79.5)	25,767 (72.3)
Mean number of stressful life events out of total 12 (mean (sd))	Missing *n* = 287	Missing *n* = 45	Missing *n* = 140	Missing *n* = 102
	1.57 (1.37)	1.89 (1.58)	1.72 (1.43)	1.36 (1.24)
How much stressful life events upset participants 1 = not too much; 3 = very much) (mean (sd))	Missing *n* = 293	Missing *n* = 46	Missing *n* = 144	Missing *n* = 103
	1.57 (1.38)	1.90 (1.58)	1.73 (1.44)	1.36 (1.24)
Stressful life events (Perceived stress) (mean (sd))	Missing *n* = 287	Missing *n* = 45	Missing *n* = 140	Missing *n* = 102
Scale 0–36	3.2 (3.13)	4.14 (3.80)	3.57 (3.31)	2.67 (2.72)
**RESOURCES**				
**non-psychological**				
**Biological**				
Absence of major illness (%)				
No	66,949 (86.5)	4581 (83.4)	31,277 (85.4)	31,091 (88.2)
Yes	10,446 (13.5)	915 (16.6)	5361 (14.6)	4170 (11.8)
Energy (mean (sd))	Missing *n* = 2415	Missing *n* = 251	Missing *n* = 1263	Missing *n* = 901
Scale 0–24 (higher score indicates more energy)	59.99 (19.88)	48.47 (22.18)	54.95 (18.84)	66.95 (18.09)
**Physical**				
Living assistance (%)	Missing *n* = 7654	Missing *n* = 613	Missing *n* = 3633	Missing *n* = 3408
No	65,564 (94.0)	4415 (90.4)	30,718 (93.1)	30,431 (95.5)
Yes	4177 (6.0)	468 (9.6)	2287 (6.9)	1422 (4.5)
Activities of daily living (mean (sd))	Missing *n* = 1265	Missing *n* = 111	Missing *n* = 641	Missing *n* = 513
Scale 1–18 (higher indicates more independence in performing activities)	17.72 (1.13)	17.44 (1.64)	17.67 (1.22)	17.82 (0.92)
Physical functioning construct (mean (sd))	Missing *n* = 4113	Missing *n* = 407	Missing *n* = 2132	Missing *n* = 1574
Scale 0–100 (higher score indicates higher health state)	68.33 (26.42)	59.63 (29.15)	64.70 (26.69)	73.36 (24.70)
**Socio-economic status**				
Education level (%)	Missing *n* = 502	Missing *n* = 47	Missing *n* = 223	Missing *n* = 232
≤HS	1930 (2.5)	228 (4.2)	1037 (2.9)	665 (1.9)
HS or GED	11,573 (15.1)	943 (17.3)	6077 (16.7)	4553 (13.0)
Vocational or training school/some college or associate degree	27,716 (36.0)	2052 (37.7)	13,290 (36.5)	12,374 (35.3)
≥College degree	35,674 (46.4)	2226 (40.9)	16,011 (44.0)	17,437 (49.8)
Annual Family Income (%)	Missing *n* = 3982	Missing *n* = 308	Missing *n* = 1972	Missing *n* = 1702
<20,000	7305 (10.0)	735 (14.2)	3839 (11.1)	2731 (8.1)
20,000–34,999	15,742 (21.4)	1278 (24.6)	7896 (22.8)	6568 (19.6)
35,000–49,999	15,650 (21.3)	1123 (21.7)	7569 (21.8)	6958 (20.7)
50,000–74,999	16,955 (23.1)	1085 (20.9)	7761 (22.4)	8109 (24.2)
75,000+	17,761 (24.2)	967 (18.6)	7601 (21.9)	9193 (2.4)
Marital Status (%)	Missing *n* = 262	Missing *n* = 19	Missing *n* = 124	Missing *n* = 119
Never married	3142 (4.1)	246 (4.5)	1472 (4.0)	1424 (4.1)
Divorced or separated	11,386 (14.8)	855 (15.6)	5137 (14.1)	5394 (15.3)
Widowed	9824 (12.7)	737 (13.5)	4699 (12.9)	4388 (12.5)
Presently married/married-like relationship	52,781 (68.4)	3639 (66.4)	25,206 (69.0)	23,936 (68.1)
**Psychological**				
**Cognition**				
Minimal Forgetfulness, (% Y)	Missing *n* = 1089	Missing *n* = 117	Missing *n* = 597	Missing *n* = 375
No	10,078 (13.2)	1442 (26.8)	6154 (17.1)	2482 (7.1)
Yes	66,228 (86.8)	3937 (73.2)	29,887 (82.9)	32,404 (92.9)
**Self and Personality**				
Personal growth construct (mean (sd))	Missing *n* = 5845	Missing *n* = 547	Missing *n* = 2856	Missing *n* = 2442
Scale 0–28 (higher score indicates higher sense of continued growth and development)	21.20 (4.96)	18.27 (5.18)	19.88 (4.80)	23.00 (4.43)
Purpose in life construct (mean (sd))	Missing *n* = 6433	Missing *n* = 612	Missing *n* = 3203	Missing *n* = 2618
Scale 0–28 (higher score indicates higher sense of purpose)	19.82 (4.78)	16.88 (4.99)	18.51 (4.51)	21.61 (4.35)
Control of beliefs (mean (sd))	Missing *n* = 117	Missing *n* = 12	Missing *n* = 61	Missing *n* = 44
Scale 0–8 (higher score indicates more internal control)	5.83 (1.81)	4.60 (1.61)	5.39 (1.67)	6.46 (1.73)
Spirituality (mean (sd))	Missing *n* = 3150	Missing *n* = 301	Missing *n* = 1506	Missing *n* = 1343
Scale 0–8 (higher indicates more religious ties)	5.68 (2.79)	5.00 (2.96)	5.41 (2.81)	6.07 (2.69)
**Social Relations**				
Social support construct (mean (sd))	Missing *n* = 4698	Missing *n* = 506	Missing *n* = 2343	Missing *n* = 1849
Scale 9–45 (higher score indicates more social support)	37.77 (7.59)	34.38 (9.12)	36.36 (7.77)	39.72 (6.58)
Living arrangements	Missing *n* = 4098	Missing *n* = 385	Missing *n* = 2078	Missing *n* = 1635
Scale 0–5 (high score indicates living with more people)	0.62 (0.54)	0.62 (0.55)	0.63 (0.55)	0.62 (0.54)
Social ties (subset of social integration) (mean (sd))	Missing *n* = 2453	Missing *n* = 242	Missing *n* = 1205	Missing *n* = 1006
Scale 1–24 (higher indicates more social ties)	18.78 (3.21)	17.70 (3.67)	18.51 (3.26)	19.24 (3.01)

## Data Availability

For further information about WHI Data Files, please visit: https://www.whi.org/page/working-with-whi-data (accessed in 2021).

## Supplementary Materials

The following supporting information can be downloaded at: https://www.mdpi.com/article/10.3390/ijerph19127089/s1, Figure S1: Plot displaying interaction between race/ethnicity and control of beliefs score as estimated from Model 5; Figure S2: Ranking of variable importance in Elastic Net model using non-psychological variables only (Top 5 variables from Model E2 shown); Figure S3: Ranking of variable importance in Elastic Net model using psychological variables only (Top 5 variables from Model E3 shown); Table S1: Description of study measures; Table S2a: Ranking of variable importance in Elastic Net model using all variables (Top 10 variables from Model E1 shown); Table S2b: Ranking of variable importance in Elastic Net model using only non-psychological variables (Top 5 variables from Model E2 shown); Table S2c Ranking of variable importance in Elastic Net model using only psychological variables (Top 5 variables from Model E3 shown); Table S3: Estimated associations between self-reported psychological resilience, energy score, and control of beliefs with race/ethnicity interactions.
