# Supplementary material for: Modifiable Resources and Resilience in Racially and Ethnically Diverse Older Women: Implications for Health Outcomes and Interventions
Source: Int J Environ Res Public Health. 2022 Jun 9;19(12):7089. doi: 10.3390/ijerph19127089 (PMC9223074; doi:10.3390/ijerph19127089)
Supplement: Supplementary file 1 [file ijerph-19-07089-s001.zip › ijerph-1733774-supplementary.pdf]

Supplemental Information

Supplemental Table S1: Description of study measures

**Outcome**	
**Resilience**	We used an adapted Brief Resilience Scale (BRS) to assess the ability to bounce back, developed by Smith and colleagues 2008. The psychometric properties were validated in four samples of adults and the authors concluded that the scale is a reliable means of assessing the degree of resilience, or the ability to bounce back or recover from stress [1]. The WHI assessed three of the six BRS questions in 2011 on form 155. Using a 5-point Likert scale (strongly disagree/agree), participants rated the following statements: "I tend to bounce back quickly after hard times," "It does not take me long to recover from a stressful event," and "I have a hard time making it through stressful events."
**Potential Modifiers**	
**Age**	Age was indicated in years at the time of screening (baseline).
**Race/Ethnicity**	Race/ethnicity was self-reported at the time of screening including<br>Black or African American, White (not of Hispanic origin), Hispanic/Latino, Asian or Pacific Islander, and American Indian or Alaskan Native.
**Stressor**	
**Stressful Life Events**	Twelve questions were used to assess stressful life events (155, v1, items 48-59.2). Participants were asked to recall if they had experienced a stressful life event in the past year and how much it upset them. For example, one item asked, "Did you have any major problems with money?" If the participant answered yes, additional response options ranged from 0= "Never" to 3= "Very Much." A higher score indicates participants experienced an increased number of stressful life events that were very upsetting. These questions originated from a modified and adapted life change measure for the Beta-Blocker Heart Attack Trial [2].
**Non-Psychological Resources**	
**Biological**	
**Absence of major illness**	Major illness and disability, stroke, diabetes, all cancers (except skin), lupus, Parkinson's disease, and Alzheimer's disease
**Energy**	Four items assessed general fatigue/energy (155, v1, items 98, 102, 104, 106). For example, one item asked during the past four weeks, "Did you feel full of pep?" Response options ranged from 0= "All of the time" to 6= "None of the time." A high score indicates a high level of energy and a lower level of fatigue. These questions originated from the 36-Item RAND Short-Form Health Survey (SF-36), developed as a part of the Medical Outcomes Study (MOS) [3].
**Physical**	
**Living Assistance**	Three items assessed whether women live in a place that provides daily assistance with everyday task (155, v1, items 29-31). These items included the following:<br>Does the place where you live have special services for older people (such as help with meals, medicines, bathing, or transportation)?<br>Did you receive any of these services?<br>In the past year, have you stayed in a nursing home?
**Activities of Daily Living**	Six questions assessed activities of daily living (155, v1, items 22-27). Item 22 asked, "Can you feed yourself?" Response options ranged from 1= "By myself" to 3=

(ADL)	"Completely unable to do this by myself." A higher score indicates more difficulty performing activities of daily living.
**Physical Functioning**	Nine questions assessed physical functioning (155, v1, items 10-19). Item 10 asked, "Does your health now limit you in vigorous activities such as running, lifting heavy boxes, or strenuous sports? And, if so, how much?" Response options range from 0="No, not at all" to 4= "Yes, limited, a lot." A higher score indicates a more favorable health state in regard to physical functioning. These questions originated from the 36-Item RAND Short-Form Health Survey (SF-36), developed as a part of the Medical Outcomes Study (MOS) [3].
**Socioeconomics**	
**Education**	Education levels were divided into four subcategories: Having attained less than a high school diploma, only having obtained a high school diploma or GED, completing some college-level courses, and obtaining a college degree.
**Income**	The income levels were divided into 4 subcategories: income of less than \$35,000, from \$35,000 to \$49,999, from \$50,000 to \$74,999, and those who made \$75,000+
**Living alone**	The following questions and options explored living conditions:<br>Answer Options for Item 29: 0 – Yes, 1 – No<br>Answer Options for Item 29.1: 1 – I live with my husband or partner, 2 – I live with my children, 3 – I live with other relatives, 4 – I live with friends, 5 – Other.<br>29) Do you live alone?<br>29.1) Who lives with you? Mark all that apply.
**Marital Status**	Marital status was divided into 2 subcategories: married-like and never married
**Psychological Resource**	
**Minimal Forgetfulness**	Symptomology scale.<br>Did forgetfulness occur? If so, was it – Mild, Moderate, or Severe?
**Personal Growth**	Seven questions assessed personal growth (155, v1, item #125-131). Item 125 asked, during the past week how true was the statement "I am not interested in activities that will expand my horizons." Response options ranged from 0= "Not at all" to 1= "Very much." A higher score indicates a higher sense of continued development. These items originated from the Psychological Well-Being scale [4, 5].
**Purpose in Life**	Seven items assessed purpose in life (155, v1, items 132-159). Item 138 asked, during the past week how true was the statement "Some people wander aimlessly through life, but I am not one of them". Response options range from 0="Not at all" to 4= "Very much." A higher score indicates a higher sense of life goals and autonomy. These items originated from the Psychological Well-Being scale [4, 5].
**Spirituality**	Two items asses spirituality (155, v1, items 148-149). Item 148 asked, during the past week how true was the statement "I found comfort in my faith or spiritual beliefs". Response options range from 0="Not at all" to 4= "Very much." A higher score indicates a higher sense of spirituality. These items originated from FACIT-Sp (Functional Assessment of Chronic Illness Therapy - Spiritual Well-Being) [6].
**Control of Beliefs**	Four questions assessed perceived stress (155, v1, items 82-85). Item 82 asked, during the past month "How often have you felt that you were unable to control the important things in your life?" Response options ranged from 1="Never to 7= "Very often." These questions originated from the Perceived Stress Scale (PSS) instrument [7, 8].
**Social Relations**	
**Social Support**	Nine items assessed social support (155, v1, items 38-46). Item 38 asked, "How often do you have someone you can count on to listen to you when you need to talk?" Response options ranged from 1= "None of the time" to 5= "All of the time." A higher score

	indicates how often nine different types of social support was available to them. These questions originated from the Medical Outcomes Study (MOS) [9, 10].
**Social Integration**	Seven possible responses to one question assessed social integration (155, v1, items 29, 29.1). Item 29: "Do you live alone?" Item 29.1 provided the following response options: (1) I live alone, (2) I live with my husband or partner, (3) I live with my children, (4) I live with my brother and sister, (5) I live with other relatives, and (6) I live with friends. A higher score indicates a higher level of social integration. These items originated from a California Human Population Laboratory, Alameda County Study [11, 12], and were modified as part of the Established Populations for Epidemiologic Studies of the Elderly (EPESE).
Note that the above information is derived from WHI form 155 (with the exception of baseline measures and demographic measures, including race/ethnicity and age). More information on form 155 and the WHI cohort can be found at the following website: https://www.whi.org/dataset/495. Additional inquires can be made at the WHI help desk at https://www.whi.org/helpdesk.	

Supplemental Table S2a. Ranking of variable importance in Elastic Net model using all variables (Top 10 variables from Model E1 shown)

Variable	Absolute value of coefficient
Control of Beliefs	0.179
Energy	0.145
Personal Growth	0.098
Mild-to-no Forgetfulness	0.091
Purpose in Life	0.089
Social Support	0.074
Stressful Life Events	0.056
Spirituality	0.037
Age	0.021
Living Arrangements	0.012

Supplemental Table S2b. Ranking of variable importance in Elastic Net model using only non-psychological variables (Top 5 variables from Model E2 shown)

Variable	Absolute value of coefficient
Energy	0.27
Stressful Life Events	0.10
Annual income 75,000+	0.03
College degree or higher	0.03
Activities of Daily Living	0.03

Supplemental Table S2c. Ranking of variable importance in Elastic Net model using only psychological variables (Top 5 variables from Model E3 shown)

Variable	Absolute value of coefficient
Control of Beliefs	0.20
Purpose in Life	0.11
Personal Growth	0.11
Social Support	0.09
No Mild-to-no Forgetfulness	0.07

Supplemental Table S3. Estimated associations between self-reported psychological resilience, energy score, and control of beliefs with race/ethnicity interactions

**Covariates**	**Model 4 (Energy)** Adjusted for stressful life events, age and race/ethnicity	**Model 5 (Control of beliefs)** Adjusted for stressful life events, age and race/ethnicity
	**Estimate (95% CI)**	**Estimate (95% CI)**
**Age**	-0.0042 (-0.0051, -0.0034); p<0.0001	-0.0055 (-0.0063, -0.0046); p<0.0001
**Race/ethnicity**	Joint p<0.0001	Joint p=0.10
Non-Hispanic White	-0.18 (-0.35, 0.0025)	-0.016 (-0.090, 0.057)
Asian or Pacific Islander	-0.35 (-0.54, -0.15)	-0.12 (-0.27, 0.024)
Black or African American	Reference	Reference
Hispanic/Latina	-0.16 (-0.33, 0.0099)	-0.12 (-0.25, 0.010)
**Stressful life events**	-0.033 (-0.034, -0.031); p<0.0001	-0.031 (-0.033, -0.030); p<0.0001
**Energy score**	0.011 (0.0089, 0.014); p<0.0001	--
**Race/ethnicity * energy score interaction**	Joint p=0.0017	
Non-Hispanic White	0.0026 (0.00014, 0.0050)	--
Asian or Pacific Islander	0.0035 (0.00065, 0.0063)	--
Black or African American	Reference	--
Hispanic/Latina	0.0014 (-0.0011, 0.0039)	--
**Control of Beliefs score**	--	0.16 (0.15, 0.18); p<0.0001
**Race/ethnicity * Control of beliefs score interaction**		Joint p=0.089
Non-Hispanic White	--	-0.0070 (-0.019, 0.0052)
Asian or Pacific Islander	--	0.0044 (-0.020, 0.029)
Black or African American	--	Reference
Hispanic/Latina	--	0.014 (-0.0085, 0.036)

Supplemental Figure S1: Plot displaying interaction between race/ethnicity and control of beliefs score as estimated from Model 5

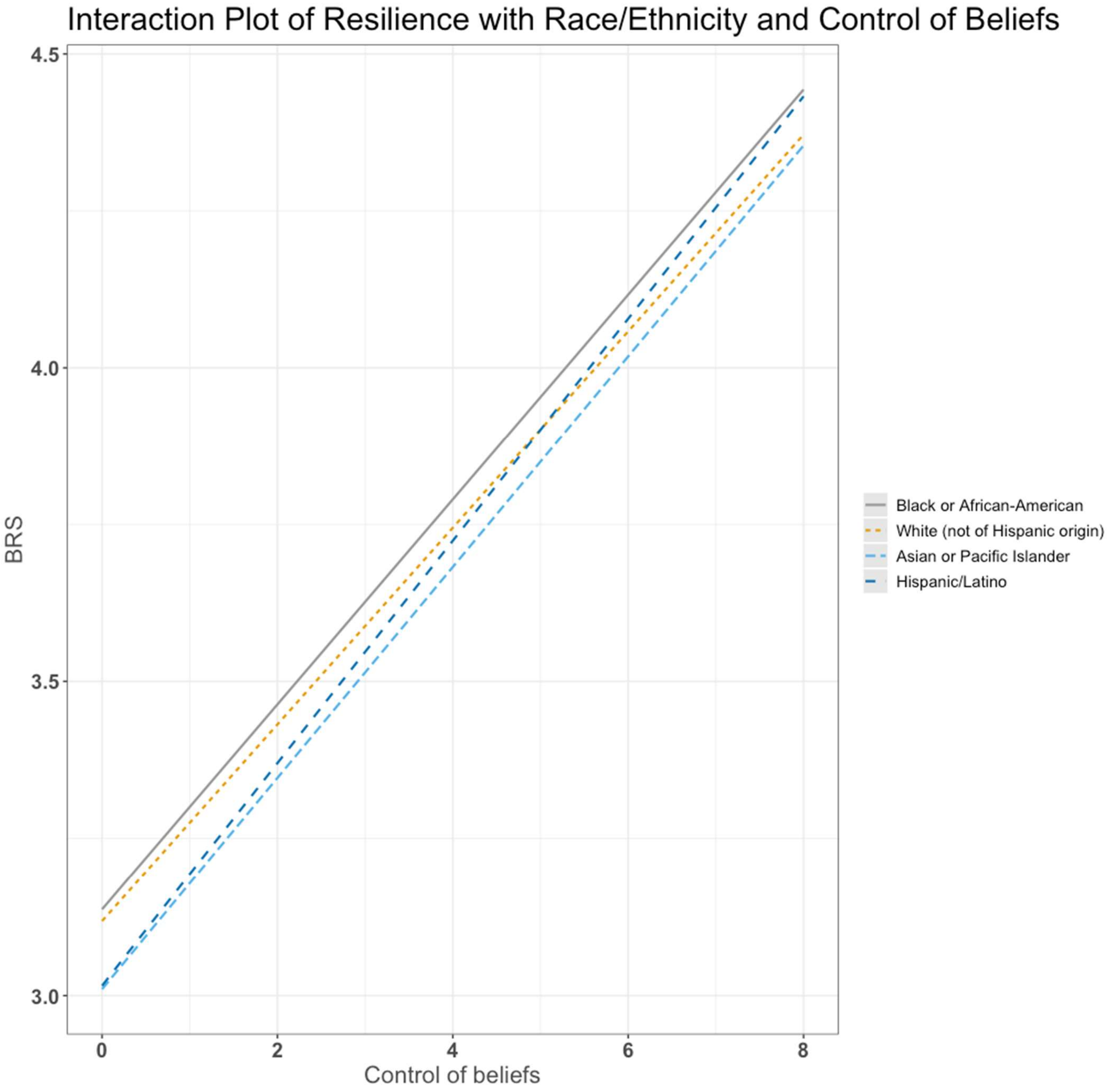

Supplemental Figure S2: Ranking of variable importance in Elastic Net model using non-psychological variables only (Top 5 variables from Model E2 shown).

Non-psychological variables only

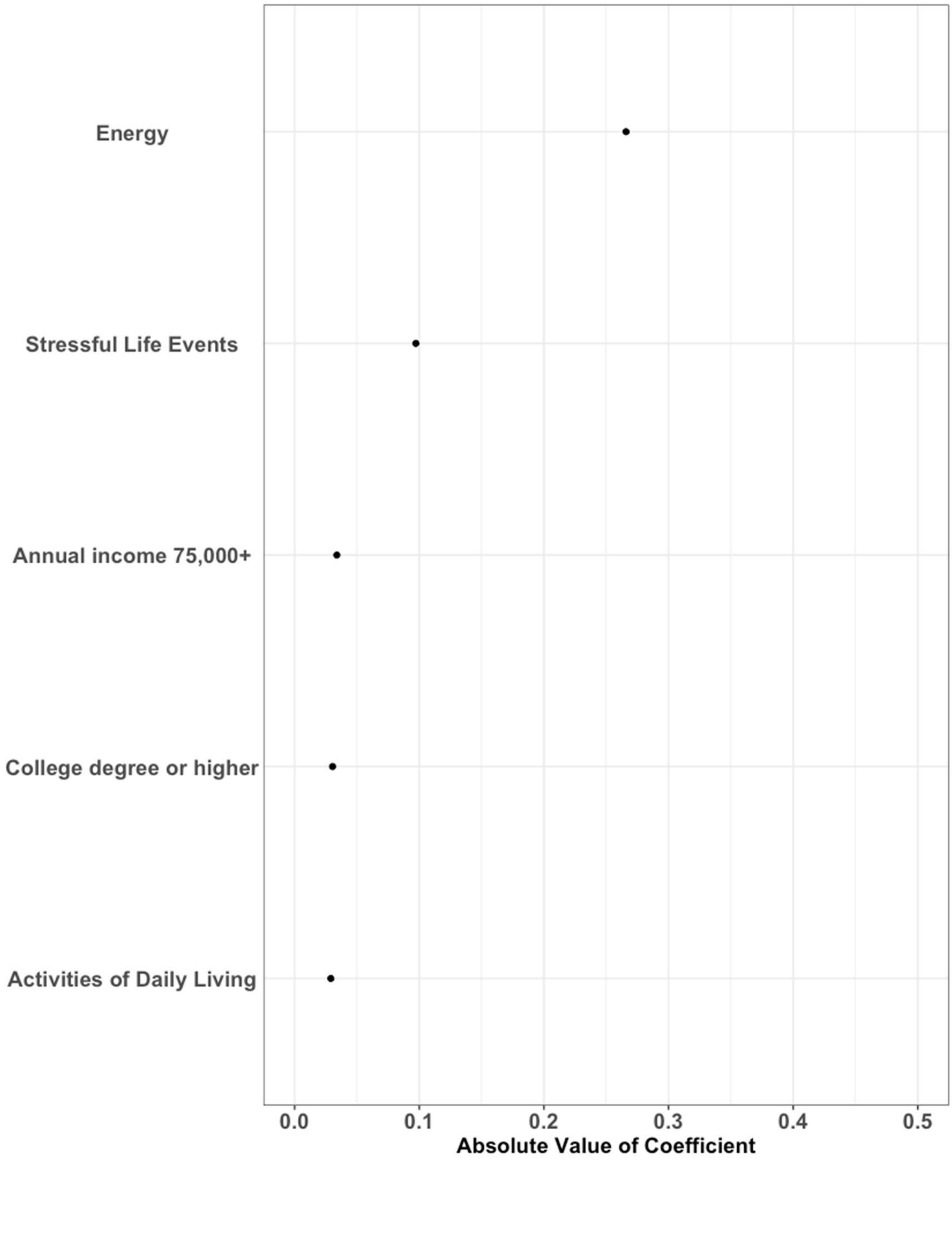

Supplemental Figure S3: Ranking of variable importance in Elastic Net model using psychological variables only (Top 5 variables from Model E3 shown)

Psychological variables only

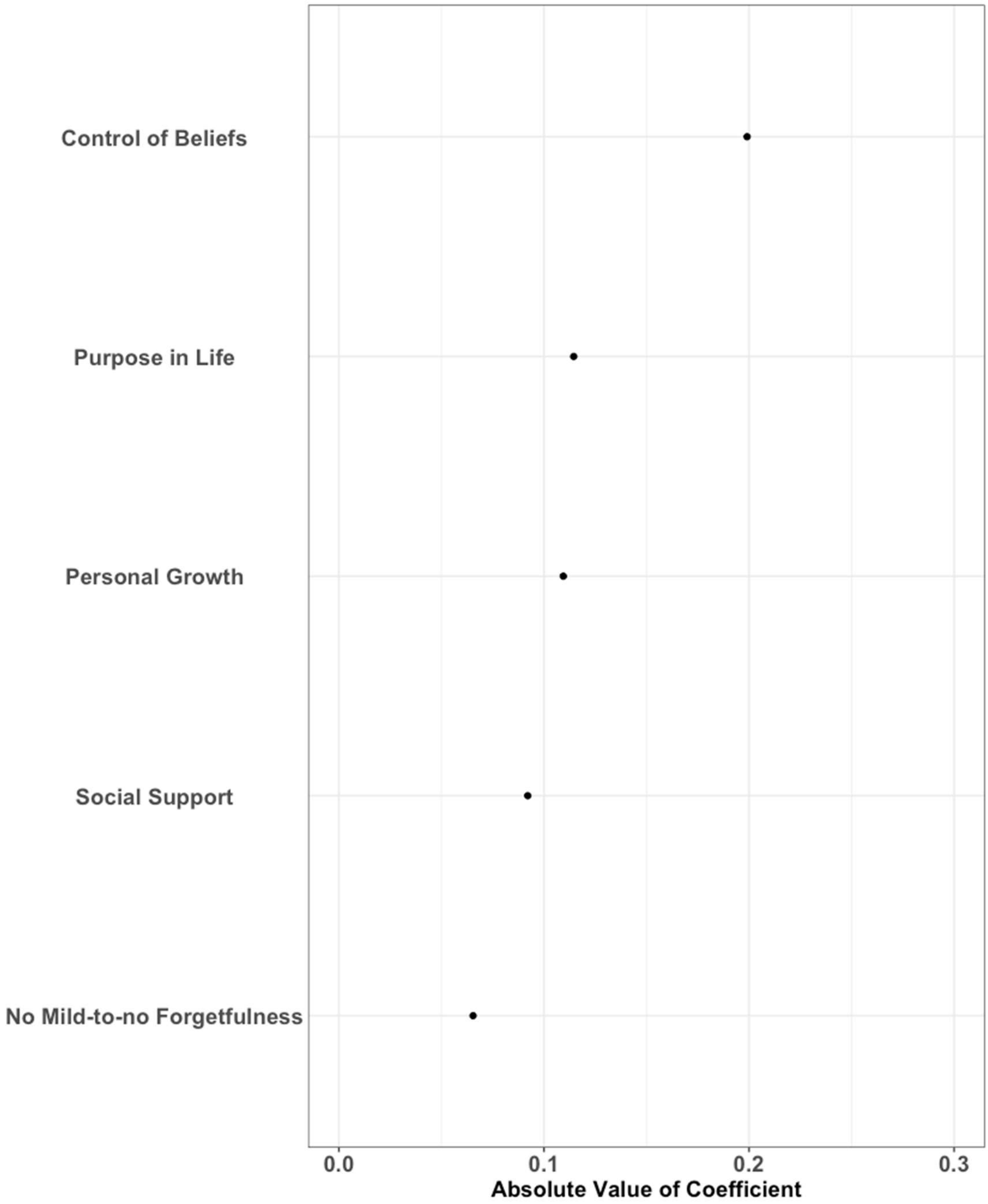

References

1. Smith, B.W., et al., *The brief resilience scale: assessing the ability to bounce back.* International journal of behavioral medicine, 2008. **15**(3): p. 194-200.
2. Ruberman, W., et al., *Psychosocial influences on mortality after myocardial infarction.* New England Journal of Medicine, 1984. **311**(9): p. 552-559.
3. Ware Jr, J.E. and C.D. Sherbourne, *The MOS 36-item short-form health survey (SF-36): I. Conceptual framework and item selection.* Medical care, 1992: p. 473-483.
4. Ryff, C.D., *Happiness is everything, or is it? Explorations on the meaning of psychological well-being.* Journal of personality and social psychology, 1989. **57**(6): p. 1069.
5. Ryff, C.D. and C.L.M. Keyes, *The structure of psychological well-being revisited.* Journal of personality and social psychology, 1995. **69**(4): p. 719.
6. Peterman, A.H., et al., *Measuring spiritual well-being in people with cancer: the functional assessment of chronic illness therapy—Spiritual Well-being Scale (FACIT-Sp).* Annals of behavioral medicine, 2002. **24**(1): p. 49-58.
7. Cohen, S., T. Kamarck, and R. Mermelstein, *A global measure of perceived stress.* Journal of health and social behavior, 1983: p. 385-396.
8. Cohen, S. and G. Williamson, *Perceived stress in a probability sample of the United States. InS. Spacapam & S. Oskamp (Eds.), The social psychology of health: Claremont Symposium on applied social psychology.* 1988, Newbury Park, CA: Sage.
9. Sherbourne, C.D. and A.L. Stewart, *The MOS social support survey.* Social science & medicine, 1991. **32**(6): p. 705-714.
10. Shumaker, S.A. and D.R. Hill, *Gender differences in social support and physical health.* Health psychology, 1991. **10**(2): p. 102.
11. Berkman, L.F., *Assessing the physical health effects of social networks and social support.* Annual review of public health, 1984. **5**(1): p. 413-432.
12. Berkman, L.F., *Social networks, support, and health: taking the next step forward.* American Journal of Epidemiology, 1986. **123**(4): p. 559-562.
